# CBP and SRF co-regulate dendritic growth and synaptic maturation

**DOI:** 10.1038/s41418-019-0285-x

**Published:** 2019-03-08

**Authors:** Beatriz del Blanco, Deisy Guiretti, Romana Tomasoni, María T. Lopez-Cascales, Rafael Muñoz-Viana, Michal Lipinski, Marilyn Scandaglia, Yaiza Coca, Román Olivares, Luis M. Valor, Eloísa Herrera, Angel Barco

**Affiliations:** 1 Instituto de Neurociencias de Alicante (Universidad Miguel Hernández - Consejo Superior de Investigaciones Científicas), Av. Santiago Ramón y Cajal s/n, 03550 Sant Joan d’Alacant, Spain; 2 Present Address: Instituto de Histología y Embriología (IHEM, CONICET/UNCuyo), Facultad de Ciencias Médicas, CC56, Universidad Nacional de Cuyo, 5500 Mendoza, Argentina; 3 Present Address: Anemocyte, Via R. Lepetit, 34 21040 Gerenzano, VA Italy; 4 Present Address: Unidad de Investigación, Hospital Universitario Puerta del Mar, Instituto de Investigación e Innovación en Ciencias Biomédicas de Cádiz (INiBICA), Av. Ana de Viya n 21, 11009 Cádiz, Spain

**Keywords:** Neuroscience, Neurological disorders

## Abstract

The CREB-binding protein (CBP) exerts tight control of developmental processes. Here, we investigated the consequences of its selective ablation in newborn neurons. Mice in which CBP was eliminated during neuronal differentiation showed perinatal death and defective diaphragm innervation. Adult-born neurons also showed impaired growth and maturation after inducible and restricted CBP loss in dentate gyrus neuroprogenitors. Consistent with these in vivo findings, cultured neurons displayed impaired outgrowth, immature spines, and deficient activity-dependent synaptic remodeling after CBP ablation. These deficits coincided with broad transcriptional changes affecting genes involved in neuronal growth and plasticity. The affected gene set included many predicted targets of both CBP and the serum response factor (SRF), an activity-regulated transcription factor involved in structural plasticity. Notably, increasing SRF activity in a CBP-independent manner ameliorated the transcriptional, synaptic, and growth defects. These results underscore the relevance of CBP–SRF interactions during neuronal outgrowth and synaptic maturation, and demonstrate that CBP plays an essential role in supporting the gene program underlying the last steps of neuronal differentiation, both during development and in the adult brain.

## Introduction

Impaired neuronal maturation, including alterations in dendrite branching and spine morphology and density, is a feature of many neurodevelopmental disorders (NDDs) [1, 2]. One of such syndromes is the Rubinstein–Taybi syndrome (RSTS, OMIM #180849) [3, 4], a congenital autosomal-dominant NDD that is caused by hemizygous mutations in *CREBBP* in most patients [5]. This gene encodes the CREB-binding protein (CBP, *aka* KAT3a), a large protein with intrinsic lysine acetyltransferase (KAT) activity that functions as a transcriptional co-activator for numerous transcription factors (TFs) [6]. Because of its dual roles as a molecular scaffold for the assembly of multimeric protein complexes at regulatory regions and a KAT for histones and other nuclear proteins, CBP is uniquely positioned to control the establishment of novel gene programs during development.

The early embryonic death of CBP null embryos at E10 due to a failure of the neural tube to close has prevented the precise examination of the role of CBP in neuronal differentiation and maturation [7]. In contrast, when CBP is eliminated in mature neurons in the postnatal brain, gross neurological defects and neurodegeneration are not observed, but restricted deficits in specific forms of memory have been reported [8–10]. The comparison of CBP hemizygous mice, which present many traits associated with RSTS [11, 12], and the comparatively milder phenotype of postnatal neuronal-restricted knockouts indicates that CBP is not only critically required during central nervous system (CNS) development [13–16], but plays a more confined role in mature neurons [17].

Here, we generated mice lacking CBP in newborn neurons by crossing *Crebbp*^f/f^ mice [18] with transgenic lines that express the Cre recombinase (either conventional or tamoxifen-regulated) under the control of the nestin promoter to precisely investigate the role of CBP in neuronal differentiation and maturation. Ex vivo and in vivo morphological analyses demonstrated that the loss of CBP in hippocampal neurons interfered with dendrite outgrowth, spine maturation, and chemically induced long-term potentiation (cLTP). Furthermore, RNA-seq data linked these phenotypes with defective activation of transcriptional programs involved in dendritogenesis, synaptogenesis, and synaptic activity. In particular, genes downstream of the serum response factor (SRF) related to neuronal growth and activity-dependent plasticity [19] were affected. This activity-regulated TF is known to participate in transcriptional complexes that contain CBP [20, 21]. Notably, the expression of a constitutively active SRF protein with the ability to transactivate target genes in a CBP-independent manner reduced the growth and transcriptional defects associated with CBP ablation. Based on these results, we conclude that CBP, and in particular its interaction with SRF, is required in newborn neurons to activate the gene program that underlies dendritic growth, spine maturation, and activity-dependent synaptic changes.

## Results

### The loss of CBP in newborn neurons causes perinatal death due to impaired diaphragm innervation

**Fig. 1:**
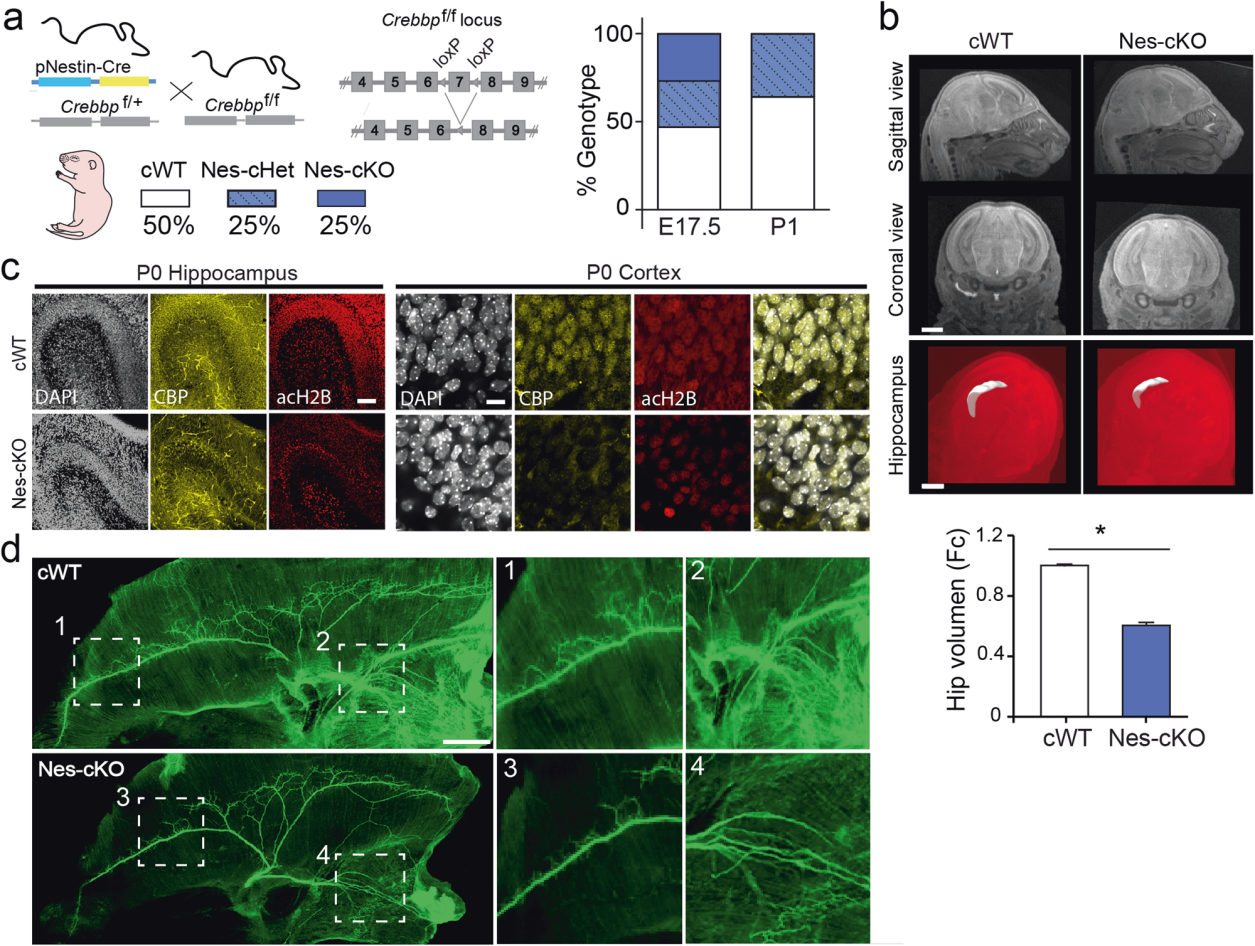
The loss of CBP in newborn neurons causes perinatal death due to innervation failure and reduced neuronal production. **a** Left panel: schematic of the genetic strategy and breeding results. Nestin-Cre:*Crebbp*^f/+^ mice were crossed with *Crebbp*^f/f^ mice; the offspring of these crosses included cWT (*no Cre:Crebbp*^*f/f*^ and *no Cre:Crebbp*^*+/f*^, both genotypes are included in the control group), Nes-cHet (*Nestin-Cre:Crebbp*^*f*/+^), and Nes-cKO (*Nestin-Cre:Crebbp*^*f/f*^) embryos. Right panel: the bar graphs show the normal Mendelian distribution of genotypes among newborns and embryos that is disrupted after birth due to the perinatal death of Nes-cKO pups (*n* = 20 litters). **b** Top: Nes-cKO pups do not show any apparent malformation in the brain. Scale bars: left panel, 1 mm; right panel, 500 μm. Bottom: hippocampal volumes were smaller in Nes-cKO pups than in control littermates (*n* = 3 pups). Student's *t* test: *P < 0.05. **c** Immunofluorescence staining showing CBP loss and histone H2B hypoacetylation in the hippocampus and cortex of Nes-cKO pups. Scale bars: 100 μm (hippocampus) and 10 μm (cortex). **d** Diaphragm innervation defects in Nes-cKO pups. Representative images of the immunostaining of the phrenic nerve innervation (right side) with anti-pan-axonal neurofilament antibody (*n* = 3 pups). Scale bar: 1 mm

We crossed CBP floxed mice (*Crebbp*^f/f^) with Nestin-cre transgenics to produce neural-specific conditional knockouts (cKOs) and investigate the consequences of selective CBP elimination in recently born neurons (Fig. 1a). Previous investigations using this Cre-driver line have reported insufficient Cre recombinase expression for gene ablation in early embryonic neural progenitors, while recombination becomes efficient during late embryonic and early postnatal periods [22]. This Cre-driver is therefore adequate to investigate the specific impact of gene ablation on newborn neurons. The offspring of these crossings were produced in the expected Mendelian ratios at birth (Fig. 1a). However, the newborn *Nestin-Cre:Crebbp*^*f/f*^ pups (hereafter referred to as Nes-cKOs) rapidly showed respiratory distress, became cyanotic and died in the first hour of life (Fig. 1a). The autopsy of Nes-cKO pups revealed pulmonary collapse, possibly due to respiratory failure, in the absence of any apparent malformation in the heart, kidneys, brain, and other major organs (Fig. 1b and data not shown). However, a 3D reconstruction of magnetic resonance images (MRI) revealed significantly smaller hippocampi in homozygous mice than in control littermates. As expected, the loss of CBP in the neurons caused a reduction in KAT activity reflected in the hypoacetylation of specific histone substrates, such as histone H2B, in the brain of P0 Nes-cKO pups (Fig. 1c). Since the autopsy suggested possible respiratory failure, we next investigated diaphragm innervation. Whole-mount diaphragms were stained with an antibody against neurofilament (NF). In control diaphragms, primary phrenic branches extended numerous secondary or intramuscular branches that formed neuromuscular junctions close to the primary branches. Motor axon projections and branches were underdeveloped in diaphragms from Nes-cKO pups (Fig. 1d), confirming that the innervation defects were a likely cause of the perinatal death. Thus, conditional CBP ablation in this strain does not interrupt embryonic development, but severely decreases survival after birth.

### Adult–newborn neurons lacking CBP also show abnormal maturation

**Fig. 2:**
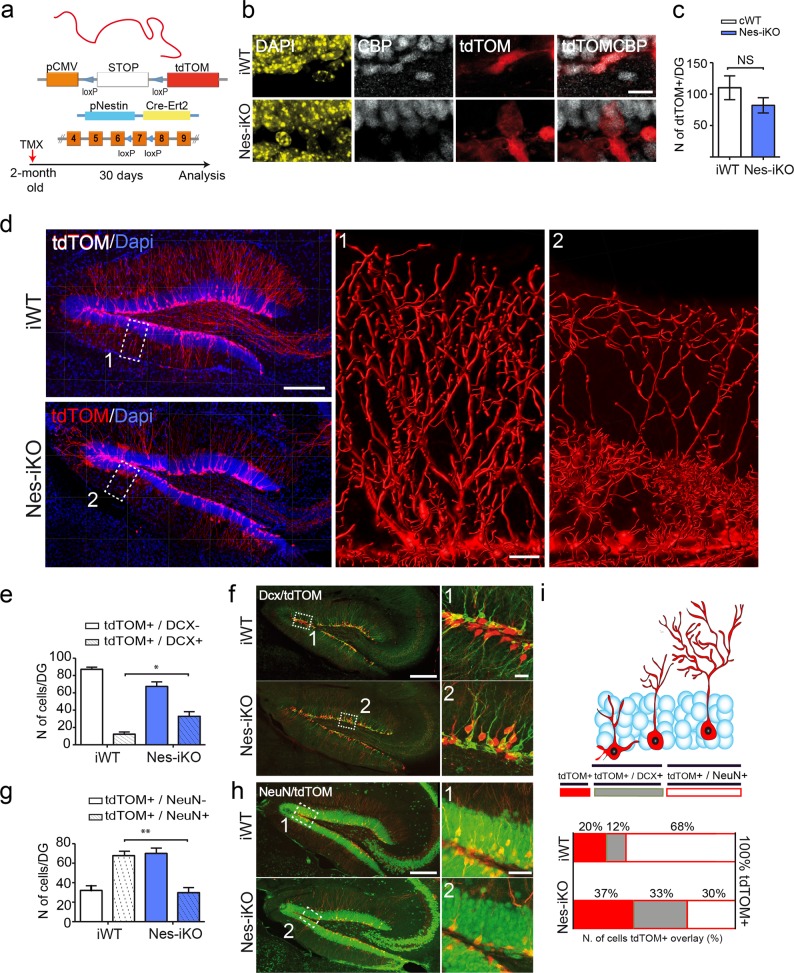
Adult-born neurons lacking CBP also show impaired growth. **a** Schematic of genetic strategy used to deplete CBP in neuroprogenitor cells in the adult brain. **b** Immunostaining with anti-CBP to verify the loss of CBP in adult-born neurons 4 weeks after TMX administration. Scale bar: 10 μm. **c** Quantification of tdTOM cells. **d** The 3D reconstruction of dendritic branches shows a dendritic defect in adult-born granular cells in the dentate gyrus of Nes-iKO mice. Scale bars: lef panel, 200 μm; right panel, 20 μm. **e**, **f** Quantification of the number of Dcx^+^ cells among tdTOM-expressing cells (**e**) and representative images of immunostaining with anti-Dcx antibodies in Nes-iKO and control littermates (**f**). Scale bars: lef panel, 200 μm; right panel, 20 μm. **g**, **h** Quantification of the number of NeuN^+^ cells among tdTOM-expressing cells (**g**) and representative images of immunostaining with anti-NeuN antibodies in Nes-iKO and control littermates (**h**). Scale bars: left panel, 200 μm; right panel, 50 μm. **i** Percentages of tdTOM-expressing cells positive for Dcx and NeuN. The upper scheme summarizes the expression of Dcx and NeuN markers during adult neurogenesis

We next investigated if CBP loss affected the maturation and outgrowth of adult-born hippocampal neurons in the same manner as embryonic-born neurons. To this end, we generated *Nestin-CreERT2:Crebbp*^*f/f*^ mice. This inducible KO strain (referred to as Nes-iKO) exhibits a loss of CBP immunoreactivity in newborn granule neurons in the dentate gyrus a few days after tamoxifen (TMX) administration. We also introduced an allele that drives the expression of the red fluorescent protein tdTomato (tdTOM) in a Cre-recombination-dependent manner to facilitate the morphological analyses (Fig. 2a). This reporter construct enables monitoring of the maturation and integration in adult neuronal circuit of newborn neurons. TMX administration in P60 mice resulted in simultaneous CBP ablation and red fluorescence-labeling of the neurons born at the time of administration; we then waited for 4 weeks for maturation of the labeled adult–newborn neurons (Fig. 2b).

Although the loss of CBP did not significantly reduce the number of tdTOM-positive (tdTOM^+^) cells (Fig. 2c), tdTOM^+^ cells displayed abnormal dendritic morphology and were mispositioned in the granule cell layer (Fig. 2d). We co-stained brain sections with the immature neuron marker doublecortin (Dcx) and the mature neuron marker NeuN to characterize these abnormal cells. More cells exhibited co-localization of Dcx and tdTOM in Nes-iKO mice than in control littermates (Fig. 2e, f), whereas the co-localization of tdTOM with the marker NeuN was reduced (Fig. 2g, h). Together, these quantitative analyses indicate a delay or inhibition of the differentiation of the newborn cells (Fig. 2i). These findings are consistent with our observations in Nes-cKO mice and underscore the importance of CBP in the last steps of neuronal differentiation, both during early postnatal development and in the adult brain.

### Cultured neurons lacking CBP show impaired dendritic growth and synaptogenesis

**Fig. 3:**
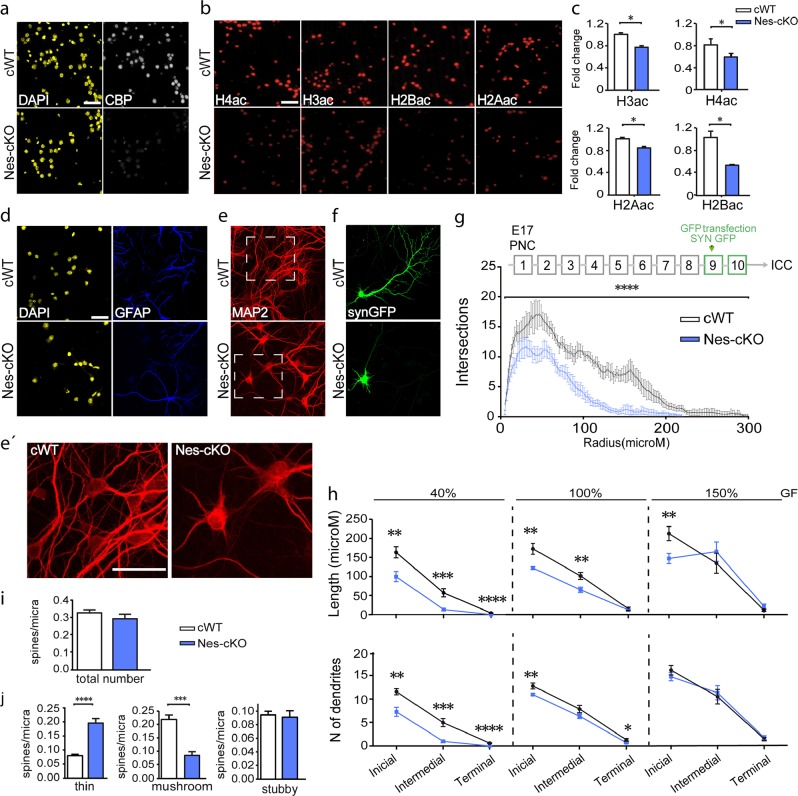
Cultured neurons lacking CBP show impaired dendritic growth and synaptogenesis. **a** Immunofluorescence staining showing CBP loss in cultured Nes-cKO hippocampal neurons at day DIV10. Scale bar: 50 μm. **b** Immunostaining for histone acetylation marks (H3ac, H4ac, H2Bac, and H2Aac) reveals a decrease in the level of acetylation in Nes-cKO PNCs at day DIV10. Scale bar: 50 μm. **c** Quantification of the immunostaining for histone acetylation in PNCs at day DIV10 (*n* = 3 independent PNCs). **d**–**f** Hippocampal neurons transfected with syn-GFP were stained for GFAP (d), MAP2 (e and e’) and GFP (f). The same fields are shown in the different pictures. Scale bar: 25 μm. **g** Sholl analysis presenting the number of intersections versus distance to soma. Error bars are shown every 2 μm. Statistically significant differences between genotypes were analyzed using two-way ANOVA (not repeated measures), ****P < 0.0001 (*n* = 4–5 neurons from three independent PNCs). **h** The concentration of trophic factors in the culture media modulates the growth defect in Nes-cKO neurons. Order range (OR): 1–8 correspond to initial dendrites; 9–16 to intermedial dendrites; and 17–32 or higher to terminal dendrites. **i**, **j** Quantification of spine density (**i**; spines/μm of dendrite length) and morphology (**j**) in Nes-cKO and control hippocampal neurons at DIV16. Student's *t* test; *P < 0.05, **P < 0.01, and ***P < 0.001; *n* = 6–8 neurons from independent PNCs

To obtain additional mechanistic insight into the role of CBP during neuronal maturation, we next investigated whether CBP loss affected neuronal outgrowth ex vivo using primary neuronal cultures (PNCs). Consistent with the atrophy observed in the MRI volumetric analysis, fewer cells were obtained from the hippocampi of Nes-cKO embryos than control littermates (numbers of cells: WT = 533 K ± 50.0; Nes-KO = 368 K ± 47.5; *n* = 7 embryos per genotype; *p* = 0.03 unpaired Student’s *t* test). Ten days after plating, an equal number of E17 hippocampal cells, immunostaining experiments confirmed the loss of CBP immunoreactivity (Fig. 3a) and the hypoacetylation of several lysine residues in the histone tails. As shown in vivo (Fig. 1c) and in several previous studies [9, 11, 23], H2B was particularly affected (Fig. 3b, c). Double staining of Nes-cKO and control cultures with antibodies against the neuron-specific cytoskeletal protein MAP2 and the astrocyte marker GFAP showed no difference in GFAP staining (Fig. 3d), whereas MAP2 staining revealed dramatic differences in neuronal morphology (Fig. 3e and e**’**). We transfected DIV9 cultures with a GFP-expressing construct under the control of the neuronal-specific synapsin promoter (syn-GFP) to precisely investigate the morphological changes occurring during the maturation of cultured neurons. After 24 h, we fixed the cells and quantified the dendrite length and number. The dendritic tree in neurons from Nes-cKO embryos was shorter and less complex than in control neurons, indicating that the ablation of CBP affects the ability of cultured neurons to elaborate a dendritic tree (Fig. 3f, g). Interestingly, these differences in neurite growth were ameliorated by increasing the concentration of trophic factors in the culture medium and aggravated upon decreasing their levels (Fig. 3h). Thus, CBP-deficient neurons may not have an intrinsic growth defect but an impaired response to neurotrophic factors.

To further explore the effects of CBP loss on hippocampal neurons, we examined synaptic density and morphology. We transfected the cultures of Nes-cKO and wild-type embryos with syn-GFP at DIV14 and waited for 48 h before cell fixation and the analysis of the morphology of dendritic spines. Although the synaptic density was similar in both genotypes (Fig. 3i), we detected an increase in the proportion of immature, thin spines (filopodia-like) in cultures from Nes-cKO embryos compared with controls, whereas the number of mature spines (mushroom-shaped) was reduced (Fig. 3j). These data indicate that synaptic maturation is also impaired in CBP-deficient neurons.

### A transcriptome analysis identifies CBP target genes in maturing neurons

**Fig. 4:**
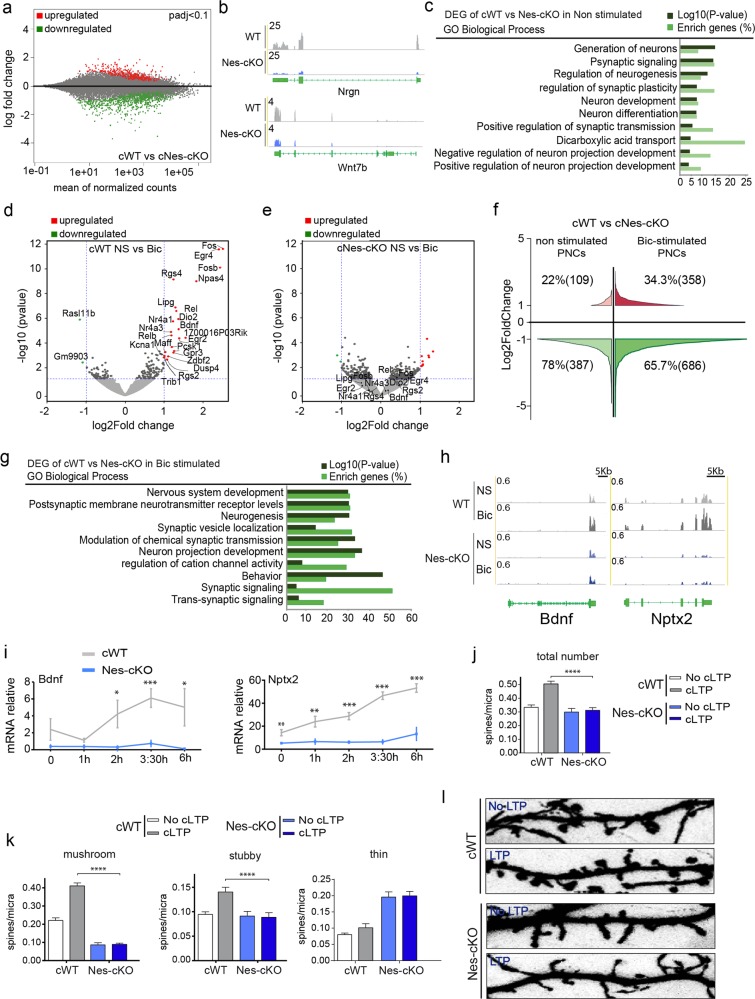
Impaired activity-induced transcription and structural changes in Nes-cKO neurons. **a** Analysis of the transcriptomes of DIV10 Nes-cKO and wild-type hippocampal cultures. Most of these changes corresponded to downregulated genes (green dots; p-adj < 0.1, abs (L2FC) > 0.3). **b** RNA-seq profiles for representative downregulated genes in Nes-cKOs. **c** Gene ontology (GO) analysis of biological processes enriched in downregulated genes in Nes-cKO PNCs under basal conditions (non-stimulated). **d** Volcano plot corresponding to DEGs between non-stimulated and Bic-stimulated (2.5 h after treatment) cortical cWT PNCs. **e** Volcano plot corresponding to DEGs between non-stimulated and Bic-stimulated (2.5 h after treatment) cortical Nes-cKO PNCs. **f** Distribution graph comparing DEGs between genotypes in non-stimulated and stimulated PNCs. **g** GO analysis of downregulated genes upon stimulation. **h** RNA-seq profiles for representative activity-regulated genes. **i** Time course analysis of the induction of selected IEG using RT-qPCR. **j**, **k** Activity-dependent synaptogenesis (**j**) and synaptic maturation (**k**) are impaired in CBP-deficient neurons. **l** Representative images of activity-dependent structural changes in spines 60 min after cLTP induction in Nes-cKO and cWT hippocampal neuronal cultures

We conducted next an RNAseq-based screen to compare the transcriptomes of Nes-cKO and control (produced from littermate embryos) DIV10 cortical PNCs and identify the transcriptional defects that underlie the phenotypes caused by CBP loss. Our stringent filtering criteria retrieved 496 differentially expressed genes (DEGs) in Nes-cKO cultures (Table S1, see Fig. S1A-B for quality controls). Consistent with the role of CBP as a transcriptional co-activator, most of the changes corresponded to downregulated genes (Fig. 4a). Among the downregulated genes, we identified genes encoding the postsynaptic protein neurogranin (*Nrgn*), the Wnt-signaling protein Wnt7b (Fig. 4b), and many other biologically relevant neuronal genes. Gene ontology (GO) analyses revealed that downregulated DEGs were related to functions, such as *Generation of neurons*, *Synaptic signaling*, *Synaptic transmission,* and *Neuron projection development* (Fig. 4c and Fig. S1C).

The large number of downregulated genes related to synaptic function suggest that the transcriptional program induced by synaptic activity may be impaired in Nes-cKO neurons. To tackle this hypothesis, we stimulated neuronal cultures with bicuculline (Bic), an antagonist of GABA_A_ receptors, which causes both presynaptic and postsynaptic activation. As expected, Bic stimulation of DIV10 cWT neurons induced the expression of immediate early genes (IEGs) involved in synaptic plasticity, such as *Npas4*, *Fosb*, and *Bdnf* (Fig. 4d and Table S2**)**. In contrast, Nes-cKO neurons were unable to trigger these transcriptional changes after Bic stimulation (Fig. 4e and Table S3). Overall, Bic stimulation exacerbated the differences between the Nes-cKO and control transcriptome both in term of number and magnitude of the changes (Fig. 4f). The negative impact on neuronal plasticity and growth pathways was particularly prominent (comparison of the data presented in Figs. 4c, g). For instance, the activity-dependent production of key plasticity-related factors, such as the brain-derived neurotrophic factor (Bdnf) and neuronal pentraxin (Nptx2) [24], was blocked in the absence of CBP (Fig. 4h).

We next conducted a time course analysis of selected target genes using independent RT-qPCR assays to evaluate the impairments in activity-driven transcription in greater detail (Fig. 4i). These experiments confirmed both the downregulation in the basal state and the deficient induction upon stimulation observed in Nes-cKO neurons in our RNAseq screen. These results are consistent with the hypothesis that CBP is actively recruited to transcriptional regulatory elements upon synaptic activation and is essential for activity-dependent neuronal maturation.

### Impaired activity-induced structural changes in Nes-cKO neurons

Since the gene sets downregulated upon CBP ablation, both at baseline and after neuronal activation, are related to synaptic functions, we next investigated whether activity-dependent synaptic maturation was impaired in CBP-deficient neurons. To address this question, we used a well-established protocol for chemical induction of long-term potentiation (cLTP), in which hippocampal neurons are exposed to a high dose of glycine for 3 min followed by washout and recovery in neuronal medium for at least 60 min [25]. As described above, we transfected the PNCs of Nes-cKO and wild-type embryos with syn-GFP 48 h before fixing the cells to visualize the morphology of dendritic spines. Afterwards, we observed a significant increase in the density of spines on wild-type neurons (Fig. 4j), primarily mushroom and stubby spines (Fig. 4k, l). However, this increase was not observed in Nes-cKO neurons (Fig. 4j, l), indicating that neurons lacking CBP are unable to undergo activity-dependent synaptic changes.

### Enhancing the SRF-dependent transcription of neuronal growth-related genes reverses the dendritic and synaptic deficits associated with CBP loss

CBP is a co-factor for many TFs regulating neuronal gene expression [6, 26]. We conducted a TF-binding site (TFBS) enrichment analysis using the promoters of genes that failed to be induced in Nes-cKO neurons after Bic stimulation to identify the transcriptional complexes that were altered in Nes-cKO neurons. This screen retrieved a number of TFs that are known to be stimulus-regulated and are involved in neuronal plasticity (Fig. S2A-B). Among these TFs, we decided to focus on SRF for several reasons: (i) this TF presented the largest enrichment in our TFBS analysis; (ii) this stimulus-regulated TF has been shown to be an essential component in the transcriptional machinery that regulates dendritic growth and synaptogenesis [27, 28] (iii) classical co-immunoprecipitation and luciferase reporter experiments [20, 29] and genomic screens [30] indicate that CBP may cooperate with SRF in binding to activity-regulated promoters and enhancers; and (iv) the amelioration of outgrowth defects in Nes-cKO PNCs by trophic factors (Fig. 3h) suggests that signal transduction cascades upstream of SRF may be involved in the deficits observed in CBP-deficient neurons.

**Fig. 5:**
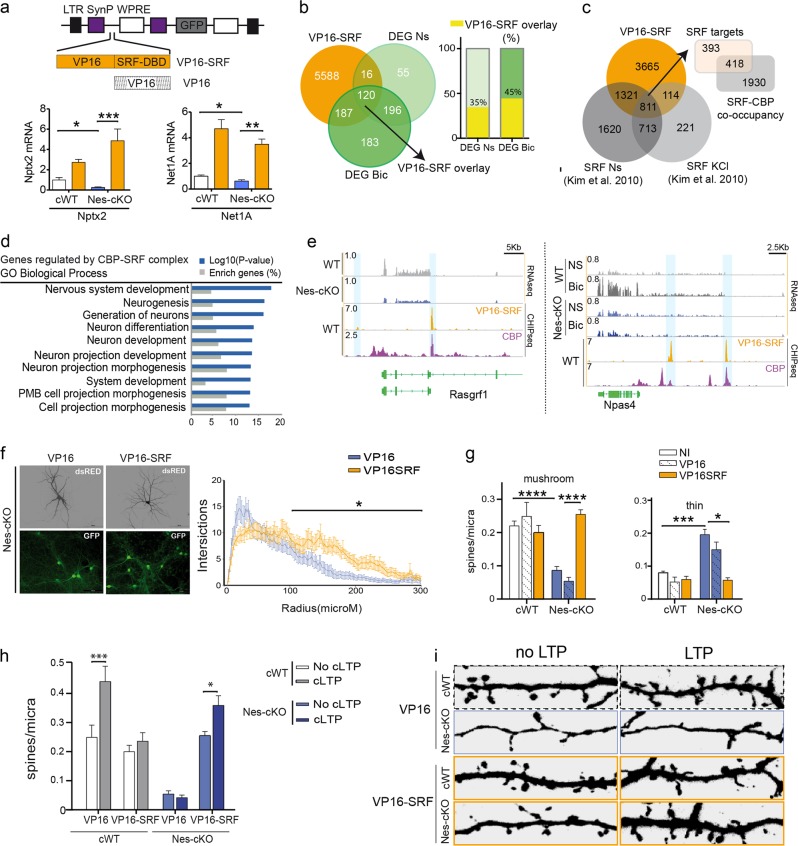
Enhancing SRF-dependent transcription of neuronal growth-related genes reverses the synaptic deficits associated with CBP loss. **a** The upper scheme shows the lentiviral (LV) constructs and color code used in the rescue experiments. LTR long-termination repeats, SynP synapsin promoter, WPRE woodchuck hepatitis virus post-transcriptional regulatory element. The lower bar graphs show RNA levels of two relevant DEGs that were upregulated upon VP16-SRF expression. **b** Venn diagram presenting the overlap between VP16-SRF target genes and DEGs in Nes-cKO neurons. The right graph shows the percentage of DEGs in non-stimulated (DEG-NS) and stimulated (DEG-Bic) PNCs that bind VP16-SRF. **c** Venn diagram presenting the overlap between VP16-SRF-bound genes and endogenous SRF binding (based on SRF occupancy profiles reported by Kim et al. 2010). The right inset shows the overlap between *bona fide* SRF targets and genes that bind to both VP16-SRF and CBP. **d** GO graph summarizing the biological processes enriched in the gene set presenting co-occupancy for both CBP and VP16-SRF. **e** IGV snapshots showing the co-localization of CBP and VP16-SRF at regulatory regions in two representative DEGs in non-stimulated (left panel) and Bic-stimulated PNCs (right panel). **f** VP16-SRF-infected PNCs were transfected with dsRed to unveil the morphology of individual neurons. The Sholl analysis revealed an increase in the length of the primary and secondary dendrites from Nes-cKO neurons infected with VP16-SRF. *P < 0.05. **g** Rescue of synaptic maturation defects in Nes-cKO neurons by VP16-SRF expresion. VP16-infected Nes-cKO neurons were used as a control. **h** Quantification of the number of mushroom spines before and after 60 min of cLTP in VP16-SRF and VP16-infected Nes-cKO neurons. **i** Representative images of activity-dependent synaptic changes in VP16-SRF and VP16-infected Nes-cKO neurons

To examine the contribution of impaired formation of CBP–SRF complexes to outgrowth and transcriptional defects, we tested whether enhancing SRF-dependent transcription in a CBP-independent manner reversed or ameliorated the deficits. Toward this end, we used a lentiviral vector that expresses a constitutively active variant of SRF, in which the VP16 transactivation domain of Herpes simplex virus (HSV) is fused to the DNA-binding domain of SRF (Fig. 5a). This chimeric protein, referred to as VP16-SRF, enables the direct recruitment of the RNA polymerase II complex to available SRF-binding sites bypassing CBP requirements [31]. Previous investigations in our laboratory have confirmed that this chimeric protein enhances the transcription of genes involved in neuronal growth [28]. Notably, 22% of the genes identified in our previous study are differentially expressed in Nes-cKO neurons, and those genes are involved in synaptic plasticity, dendritogenesis, and neurogenesis (Fig. S2C).

To assess the effect of VP16-SRF expression on CBP-deficient neurons, we first examined whether the expression of relevant DEGs downstream of SRF was enhanced by VP16-SRF. RT-qPCR experiments confirmed their upregulation in both control and Nes-cKO neurons (Fig. 5a). Next, to investigate the mechanism underlying this reversal, we determined the genomic occupancy profile of VP16-SRF in hippocampal neurons by ChIP-seq using an antibody that recognizes the VP16 domain. VP16-SRF binds to more than 10,000 regions in hippocampal chromatin, which are associated with 5911 genes (Table S4) and are preferentially located at distal intergenic and intronic regions (Fig. S2D). Notably, 85% of the genes associated with VP16-SRF peaks also showed CBP occupancy according to the CBP-binding profile in hippocampal chromatin produced in our laboratory (Fig. S2E), a percentage of overlap much larger than the one expected by chance (*p*-value = 7.44 × 10^−69^, exact hypergeometric probability test). Moreover, VP16-SRF and CBP bind at the same genomic location in 2348 genes (47% of co-occupied genes; Fig. S2F). This high level of coincidence suggests that these genes are co-regulated by SRF and CBP. Consistent with this hypothesis, many of the genes deregulated in CBP-deficient neurons (35% of DEGs in non-stimulated neurons and 45% of DEGs in Bic-stimulated neurons) showed VP16-SRF binding (Fig. 5b). We next compared the ChIP signal for VP16-SRF with genomic occupancy profiles for SRF in non-stimulated and in KCl-stimulated primary cortical cultures [30] to test if this chimeric protein binds to the same sites as the endogenous SRF. Despite the very low genomic coverage in the seminal ChIP-seq experiments by Kim et al., we observed a large overlap with our VP16-SRF profiles under both conditions (48% in non-stimulated cells and 50% in stimulated cells; Fig. 5c). The intersection of the three ChIP-seq profiles was occupied by 811 genes, which we consider *bona fide* targets of SRF in cultured neurons. Supporting the hypothesis that the deregulation of genes dependent on CBP–SRF complexes is responsible for the phenotype of Nes-cKO neurons, 418 *bona fide* targets of SRF displayed overlapping VP16-SRF and CBP binding (Fig. 5c, inset). Furthermore, this gene set was highly enriched in genes involved in biological processes, such as neuron development and differentiation, and neuron projection development and morphogenesis, which are strongly correlated with the reported phenotypes (Fig. 5d). Some examples of DEGs that are involved in these processes and co-regulated by CBP and SRF in non-stimulated and Bic-stimulated PNCs are shown in Fig. 5e and S2G-H.

Finally, we tested whether the CBP-independent enhancement of SRF transcription ameliorated or reversed the outgrowth defect in Nes-cKO neurons to directly assess the relevance of the gene program downstream of SRF–CBP complexes. Sholl analyses revealed an increase in the length of intermediate and terminal dendrites of Nes-cKO neurons after infection with VP16-SRF compared with VP16 control (Fig. 5f). Notably, infection with VP16-SRF also restored the number of mature and intermediate spines and simultaneously decreased the number of immature spines (Fig. 5g). Furthermore, activity-dependent synaptic deficits were also reduced. Thus, the number of mushroom spines observed after 60 min of cLTP was increased in Nes-cKO neurons after infection with VP16-SRF compared with controls (Fig. 5h, i). Based on these results, the SRF–CBP transcriptional complex plays a critical role during neuronal maturation by regulating processes, such as dendritogenesis, synaptogenesis, and activity-depending synaptic remodeling.

## Discussion

CBP is known to regulate cell lineage differentiation during early development [32]. At later stages, it is also involved in regulating the differentiation of a variety of neural cell types, such as motoneurons, cortical progenitors, and astrocytes [14, 16, 17, 33, 34]. However, the consequences of its elimination in forebrain principal neurons at the time of neuronal differentiation and maturation had not been explored.

Nes-cKO mice represent an ideal model to investigate the role of CBP during neuronal differentiation and maturation in vivo. These mice showed a reduced hippocampal volume, impaired dendritic growth, and immature synapses with concomitant hypoacetylation at nucleosome histones, indicating that CBP is essential at this stage of neuronal development. The experiments in Nes-iKO mice and CBP-deficient PNCs further confirmed this view and revealed that the loss of CBP also interfered with the expression of mature neuron markers in the adult brain and in cultured neurons. Underscoring the clinical relevance of these findings, a recent study has reported the abnormal morphology of neurons derived from induced pluripotent stem cells (*aka* iNeurons) from selected patients with RSTS (who bear mutations in either *CREBBP* or *EP300*), although the magnitude of the anomalous neuronal morphology varied among patients [4]. The results presented here, together with previous findings by other researchers and our group, indicate that the cognitive and neurological deficits associated with RSTS [11] have three distinct components: the first one originates during the embryonic development of the CNS and is related to the epigenetic programming of cell identity [35]; the second is derived from the impaired or delayed neuronal maturation, as reported here; and the third results from the continuous requirement for the enzymatic activity of CBP throughout life [8–10, 36].

We specifically assessed here the impact of enhancing SRF-driven transcription to rescue the second type of deficit. Several lines of evidence support a functional interaction between SRF, an important regulator of adaptive responses that involve changes in neuronal morphology [19], and CBP. For instance, studies in mice with a conditional loss of SRF in neurons revealed defects in LTP, but no impairment in neuronal survival [37, 38], which is also observed in CBP-deficient mice [11]. Co-immunoprecipitation experiments and co-transfections of CBP with serum-responsive reporter plasmids indicate that CBP cooperates with SRF and suggest that both proteins are part of the same multimolecular complex [20]. The physical interaction between SRF and CBP has been further confirmed by two-hybrid assays in mammalian cells [21]. Moreover, SRF-mediated induction of the IEG Fos is potentiated by the recruitment of CBP by the ternary complex factor Elk-1 [29]. Based on these findings, CBP was proposed to constitutively bind to the SRE in a higher order complex, which would facilitate the rapid transcriptional activation [29]. Our results are consistent with this model and show for the first time the relevance of SRF–CBP interactions in newborn neurons. According to our results, CBP–SRF transcriptional complexes play an essential role in the late stages of neuronal maturation by affecting dendritogenesis, synaptogenesis, and activity-dependent synaptic maturation. Moreover, our results suggest that strategies designed to enhance SRF signaling in early postnatal stages represent a potentially promising therapeutic approach for preclinical models of RSTS by intervening in the impaired CBP-dependent neuronal growth and synaptic maturation.

## Materials and methods

### Mouse strains

*Crebbp*^f/f^ [18], CMV-fxSTOPfx-tdTomato (*tdTOM*) [39], *Nestin-Cre* [40], and *Nestin-Cre-ER*^*T2*^ [41] strains have been described previously. The last three strains are available at the Jackson laboratory with the stock numbers #7914, #3771 and #16261, respectively, while the first strain was provided by Beat Lutz’ lab (Institute of Molecular Biology, Mainz, Germany). Note that both *Nestin-Cre* and *Nestin-Cre-ER*^*T2*^ mice express the Cre recombinase under the control of the Nestin promoter, but in the second case recombination is controlled by TMX-induced nuclear translocation of a chimeric recombinase [41]. In the experiments using *Nestin-Cre-ER*^*T2*^, recombination was triggered by TMX administration when the mice were 8-week-old (intragastric delivery of 200 mg/kg dissolved in 100% corn-oil for 5 alternative days); and mice were perfused 4 weeks after TMX administration. The genetic background of all mice is C57BL/6 J, except for the ChIP-seq experiment conducted in PNCs from wild-type ICR embryos. Experiments were conducted blind, and genotypes were provided for statistical analyses. No animals were excluded from the study. Every animal was used in a single experiment. All the mice were maintained and bred in pathogen-free conditions within the animal house at the Instituto de Neurociencias (UMH-CSIC) with a 12-h light/dark cycle, constant temperature (22 ± 1 °C) and relative humidity (55 ± 5%), with free access to food and water. Experimental designs were consistent with Spanish (BOE 34/11370-421, 2013) and European Union Council (2010/63/EU) regulations and approved by the Institutional Animal Care and Use Committee.

### Neuronal culture preparation and treatments

Primary hippocampal and cortical neurons were obtained from E17.5 embryos. Hippocampus and cortex were dissected and processed as described previously [31]. In the experiments with Nestin-cKO and cWT PNCs, each embryo was processed separately. In the experiment with ICR embryos (ChIP-seq), all embryos were pooled together before plating the cells. The day of plating was considered day in vitro 1 (DIV1). In the rescue experiment with B-27™ Supplement (Gibco), the culture media used through the whole experiment was prepared with different concentrations of this reagent. In stimulation experiments, DIV10 cortical cultures were exposed to a mix of bicuculline (25 μM) and AP-4 (1 mM) for the indicated times. Chemical long-term potentiation (cLTP) was induced in DIV16 hippocampal cultures as described previosly [25]. In the experiments involving lentivirus (LV) infection, hippocampal and cortical neurons from Nestin-cKO embryos were infected at DIV3 and fixed for immunofluorescence analysis or collected for RNA isolation, respectively, at DIV10. LV-syn-VP16 and LV-syn-VP16-SRF were produced as described previously [31]. In Sholl analysis experiments, the plasmid pDsRed-Express2-C1 (Clontech) driving dsRed expression or pSyn-GFP driving GFP expression were transfected using Lipofectamine 2000 (Invitrogen) at DIV9 and the cultures were fixed 1 day later. In cLTP experiments, the plasmid pDsRed-Express2-C1 (Clontech) driving dsRed expression were transfected using Lipofectamine 2000 (Invitrogen) at DIV14 and the cultures were fixed at DIV16 for spine morphology analysis.

### Immunocytochemistry, immunohistochemistry, and image analysis

PNCs grown on glass coverslips were fixed and stained with 4% paraformaldehyde in PBS (12 min), washed in PBS and PBS−0.1% Triton X-100 (PBT) and incubated for 0.5 h at room temperature with 3% newborn calf serum (NCS)-PBT, as previously described [28]. Coverslips were incubated overnight at 4 ^o^C with the primary antibodies diluted in 2% NCS-PBT. The primary antibodies used in immunocytochemistry are α-MAP2 (Sigma, M9942), α-GFAP (Sigma, G9269), α-GFP (Aves Labs, GFP-1020, valid also for recognizing EYFP), α-CBP (A22, Sigma sc369), α-AcH2A (K5, 9, 13, 15) (Sigma, 07-376), α-AcH4 (K5, 8, 12, 6), α-AcH3 (K9, 14), and α-AcH2B (K5, 12, 15, 20) (generated in Barco’s lab [42]). In immunohistochemistry experiments, mice were perfused with 4% paraformaldehyde in PBS as described previosly [28]. Coronal vibratome sections (80-μm) were obtained from the brain of *Nestin-CreERT2:Crebbp*^*f/f*^*:tdTOM* and *Nestin-CreERT2:tdTOM* mice perfused 4 weeks after TMX administration. Brain sections were washed in PBS and PBT and incubated for 30 min at room temperature with 3% NCS-PBT. The primary antibodies used in immunohistochemistry are α-dsRed (Clontech, 632496), α-Dcx (ab18723), and α-NeuN (Chemicon MAB377). Neuronal cultures and brain slices were both counterstained with a 1 nM DAPI solution (Invitrogen) before mounting. Photos were taken with an Olympus confocal inverted microscope or a Leica epifluorescence microscope, except for the diaphragm images that were taken with a Leica fluorescence stereomicroscope. For whole-mount staining of mouse diaphragms, P0 pups were perfused with 4% paraformaldehyde in PBS for 2 min. Diaphragms were then dissected and washed in 0.1 M glycine in PBS, blocked overnight in blocking buffer (0.5% Triton X-100, 3% BSA in PBS) at 4 °C, and incubated overnight in blocking buffer with primary α-Pan-axonal neurofilament (1:1000, Covance #SMI-311R) also at 4 °C. After several washes, samples were incubated overnight at 4 °C in blocking buffer with an Alexa-488 anti-mouse antibody (1:500, Jackson ImmunoResearch). In the cell counting experiments in Nes-iKOs, brain slices were consecutively numbered and four sections (taken 80 μm apart) per animal were randomly selected. Confocal scans were taken using the 20x objective (2-μm z-step; area = 0.72 mm [2] per field containing the whole dentate gyrus, in total 0.32 mm^3^, and keeping constant pinhole, contrast, and brightness). Images were converted to green or red and analyzed with ImageJ software. To calculate the percentage of tdTOM/NeuN and tdTOM/Dcx double-positive cells, all tdTOM cells in the granule cell layer and subgranular zone in the z-stacks were counted. The number of double-positive cells from four sections per mouse was divided by the number of tdTOM cells in the same sections to calculate a percentage value for each mouse (*n* = 3 mice per genotype). The images shown are reconstructions in z-stack with the average intensity. For the analysis of spine density and size, the program NeuronStudio was utilized as previously described [25]. For Sholl analysis, the dendrites of transfected neurons were drawn and analyzed with the Neurolucida tracing Software (MBF Bioscience) in a blind manner. The number of intersections was determined every 2 μm, starting at a radius of 5 μm from the center of the neuronal soma. For the analysis of the length and number of dendrites (Fig. 3h), we grouped the data into order ranges: order 1–8, initial dendrites; order 9–16, intermedial dendrites; and order 17–32 (or higher), terminal dendrites. The level of histone acetylation in the PNCs was quantified by intensity of fluorescence with ImageJ software after marking the nucleus area as region of interest (ROI).

### MRI analyses

MRI analysis of P0 Nestin-cKOs and *Crebbp*^*f/f*^ pups was carried out in a horizontal 7T scanner with a 30-cm diameter bore (Biospec 70/30 v, Bruker Medical, Ettlingen, Germany) as previously described [43] with some modifications. T2-weighted anatomical images were collected using a rapid acquisition relaxation enhanced sequence (RARE), applying the following parameters: 15 slices, 30 × 30 mm field of view (FOV), 1-mm slice thickness, 256 × 256 matrix, 56 ms effective echo time (TEeff), 2 s repetition time (TR), and 8 RARE factor. The B0 field distribution in a large voxel (40 × 40 × 40 mm^3^) containing the volume to be imaged was acquired (FieldMap). Samples were localized with a T2-weighted RARE sequence, and first- and second-order shims adjusted with MAPSHIM application in a sufficiently large voxel containing the brain. Three-dimensional data were acquired using a RARE 3D sequence with 1500 ms TR, 46.8 ms TEeff, 16 RARE factor, and 16 averages in a total acquisition time of 13.6 h. The matrix size was 300 × 200 × 170 using a FOV of 30 × 20 × 17 mm^3^, which yielded an isotropic resolution of 100 μm. Data were acquired and processed with Paravision5.1 software. Image resolution was digitally increased by linear interpolation rendering a final isotropic resolution of 30 μm. Length and volume measurements were performed using ImageJ software. Three-dimensional reconstructions of MRI images were created with Imaris software.

### RNA extraction and RT-qPCR

Total RNA from tissue and hippocampal cultures was extracted with TRI reagent (Sigma-Aldrich) and reverse transcribed to cDNA using the RevertAid First-Strand cDNA Synthesis kit (Fermentas). qPCR was performed in an Applied Biosystems 7300 real-time PCR unit using Eva Green qPCR reagent mix. The primer sequences used in RT-qPCR assays are: *Nptx2*, forward: 5ʹ-GAACGCCCTGCTGCAGAGv-3ʹ, reverse: 3ʹ-AATGCACTGTTGCCTCGCT-5ʹ; *Bdnf*, forward: 5ʹ-AAGTCACACCAAGTGGTGGGC-3ʹ, reverse: 3ʹ-GGATGGTCATCACTCTTCTCACCT-5ʹ; *Gapdh*, forward: 5ʹ-CTTCACCACCATGGAGAAGGC-3ʹ, reverse: 3ʹ-CATGGACTGTGGTCATGAGCC-5ʹ.

### RNA-seq, ChIP-seq, and bioinformatical analyses

Cortical PNCs of 2 cWTs and 3 Nes-cKOs embryos were treated or not with Bic and sequenced separately. The 10 samples were sequenced according to the manufacturer instructions in a HiSeq 2500 sequencer (Illumina, Inc). Briefly, RNA-seq reads were mapped to the mouse genome (Mus_musculus.GRCm.38.83) using STAR (v2.5.0c) [44]. Quality control of the raw data was performed with FastQC (http://www.bioinformatics.babraham.ac.uk/projects/fastqc/). Library sizes were between 19 and 26 million single reads. Analysis and preprocessing of data were performed using R (v3.4.3“Kite-Eating Tree”) statistical computing and graphics. Samtools (v1.3) were used to handle BAM files [45]. To retrieve differentially expressed genes (DEG), mapped reads were counted with HTSeq v0.6.1 [46] and count tables were analyzed using DeSeq2 v1.10.0, R-package [47] and bioconductor v3.2 (BiocInstaller 1.20.3). Genes were considered differentially expressed at Benjamini–Hochberg (BH) adjusted *p*-value < 0.1 and absolute log_2_-fold change > 1. Significantly upregulated and downregulated genes were visualized with IGV (v2.3.72) and clustering analysis. Chromatin immunoprecipitation (ChIP) from PNCs was performed as previously described using an anti-VP16 (Sigma V4388) antibody [43]. ChIP-seq was performed in a HiSeq 2500 machine using Flow Cell v4 in single-end configuration (50 bp). Quality control of the raw data was performed with FastQC (http://www.bioinformatics.babraham.ac.uk/projects/fastqc/). Libraries were trimmed from adapters and low-quality bases using Trimmomatic v0.36 [48], and mapped to reference genome (Ensembl GRCm38) using Bowtie2 v2.3.2 [49]. Data were further processed using samtools (v1.5) [45] and bedtools (v2.26.0) [50]. Peak calling for ChIP-seq datasets was performed using MACS2 (v2.1.1) [51], SeqMINER [52] was used to generate the k-means clustering data and the merged ChIP profiles. Further data processing was performed with custom scripts in the R programming language (https://cran.r-project.org/). Data from ref. [30] were lifted over from mm9 to mm10 using the R package rtracklayer. GO enrichment analyses were performed using the platform Panther [53]. TFBS enrichment analysis was conducted using the Enrichr website (http://amp.pharm.mssm.edu/Enrichr/).

### Statistical analyses

Statistical analysis was performed using Prism 6.0 software (GraphPad). Sample size was decided based on previous experience and the literature in this field. Comparison between animals/pups are based on genotype, and no randomization method was applied. In PNC experiments using different treatments, wells were randomly assigned to the various experimental conditions (unbiased protocol). Two-tailed Student’s *t* tests were used to compare the means between the samples and their respective controls. In Sholl analyses, the statistical difference between genotypes were assessed by two-way ANOVA (non-repeated measures). For pairwise comparison of averages data were tested for normality. If any of the two samples were significantly non-normal, a non-parametric Mann–Whitney U/Wilcoxon rank-sum test was executed instead. If the data from the two samples met the normality criterion, an F test was used for equality of variances. The *P*-values of Student’s *t* tests and two-way ANOVA are represented in the figures by asterisks (*P < 0.05, **P < 0.01, ***P < 0.001 and ****P < 0.0001). The absence of asterisk indicates that the change relative to control was not statistically significant.

## Supplementary information

Supplemental figures and tables

Supplementary Tables

## Data Availability

The RNA-seq and ChIP-seq datasets are available at the GEO Series GSE118248.
