# Supplementary material for: CBP and SRF co-regulate dendritic growth and synaptic maturation
Source: Cell Death Differ. 2019 Mar 8;26(11):2208–22. doi: 10.1038/s41418-019-0285-x (PMC6889142; doi:10.1038/s41418-019-0285-x)
Supplement: Supplementary file 1 — Supplemental figures and tables [file 41418_2019_285_MOESM1_ESM.pdf]

Supplemental Figures

Supplemental Figure S1 Impaired activity-induced transcription and structural changes in Nes-cKO neurons. **A.** IGV snapshot of the *Crebbp* locus and quantification of the number of reads mapping to exon 7. The difference between Nes-cKO and cWT neurons confirms the specific ablation of exon 7 in PNCs from Nes-cKO embryos. **B.** Correlation between RNA-seq samples. Colors represent the Pearson correlation coefficients (R2), from low (0) to high (80) correlation. **C.** Box plot depicting the total reads into genes belonging to the indicated biological processes. *P* values were determined using the Wilcoxon non-parametric test.

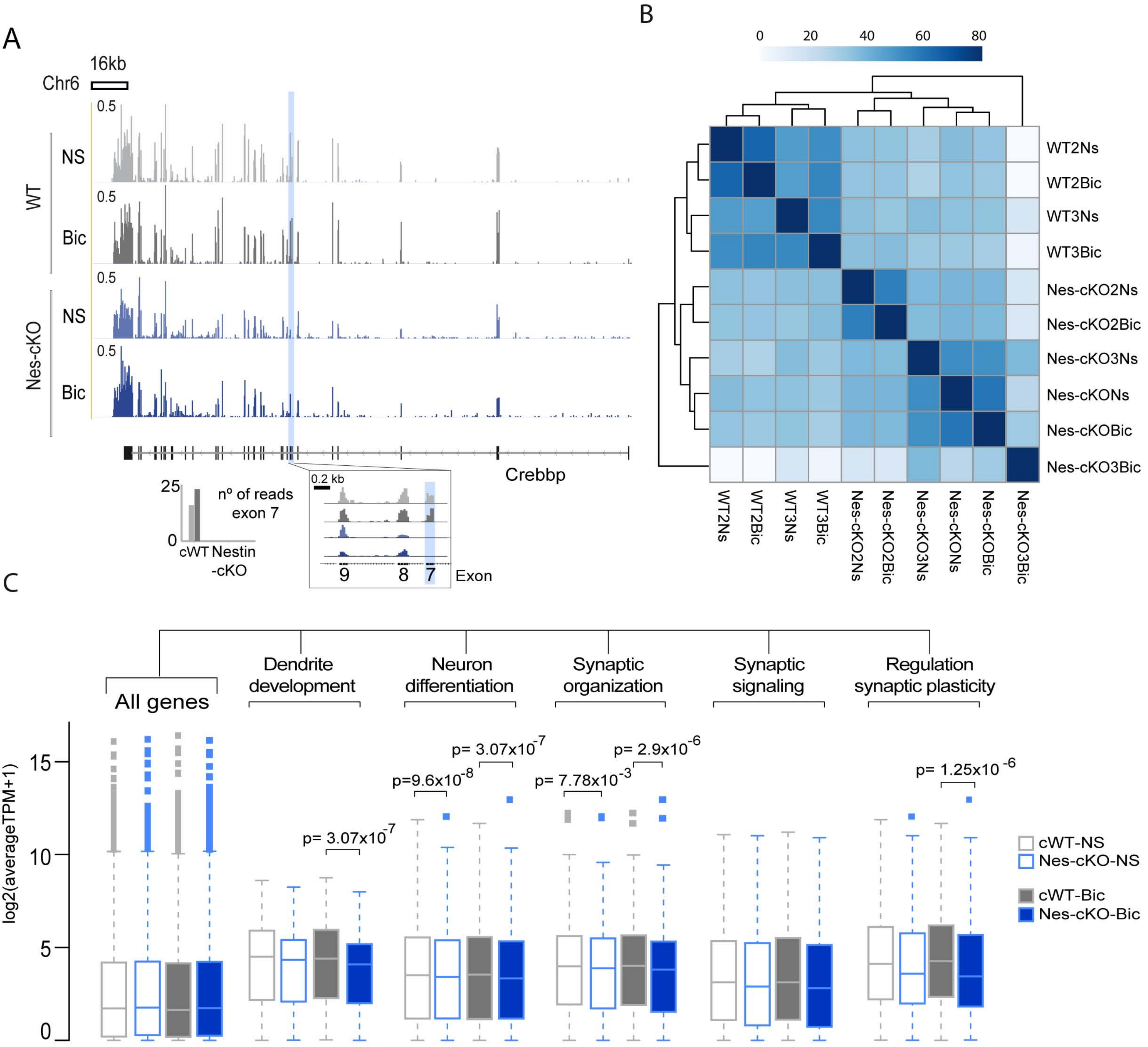
A
B
C
16kb
Chr6
WT
NS
Bic
Nes-cKO
0.5
Crebbp
0.2 kb
nº of reads
exon 7
25
0
cWT
Nestin
-cKO
9
8
7
Exon
0
20
40
60
80
WT2Ns
WT2Bic
WT3Ns
WT3Bic
Nes-cKO2Ns
Nes-cKO2Bic
Nes-cKO3Ns
Nes-cKONs
Nes-cKOBic
Nes-cKO3Bic
All genes
Dendrite development
Neuron differentiation
Synaptic organization
Synaptic signaling
Regulation synaptic plasticity
log2(averageTPM+1)
p= 3.07x10-7
p=9.6x10-8
p= 3.07x10-7
p= 7.78x10-3
p= 2.9x10-6
p= 1.25x10 -6
cWT-NS
Nes-cKO-NS
cWT-Bic
Nes-cKO-Bic

Supplemental Figure S2 related to Figure 5. Enhancing SRF-dependent transcription of neuronal growth-related genes reverses the synaptic deficits associated with CBP loss. A. TFBS-enrichment analysis based on UCSC Genome Browser position weight matrices (PWMs) identifies SRF as the TF showing the largest enrichment in the promoters of downregulated genes in Nes-cKO neurons after Bic-stimulation. **B.** TFBS-enrichment analyses based on TF Chip-seq data generated in the ENCODE project identifies SRF as the TF showing the most significant enrichment. **C.** Genes upregulated by VP16-SRF (left bar graph) and downregulated in CBP-deficient neurons are related to neuronal growth and plasticity according to GO enrichment analyses (right sector graph). **D.** Mapping of VP16-SRF binding in hippocampal neurons. The sector diagram shows the distribution among gene features. **E.** Sector graph representing the percentage of genes annotated with both VP16-SRF and CBP peaks (85%), or only VP16-SRF peaks (15%). The distance thresholds for annotation to a given gene are 5 Kb upstream and 1 Kb downstream. **F.** The kernel plot depicts CBP (purple) at VP16-SRF (orange) occupancy peaks. The CBP distribution is represented upstream (+ 2 Kb) and downstream (-2Kb) of the center of the VP16-SRF peak. Blue: input for the CBP sample; gray: input for the VP16-SRF sample. **G-H.** Additional IGV snapshots of DEGs in non-stimulated (G) and Bic-stimulated PNCs (H) showing the co-localization of CBP and VP16-SRF at regulatory regions of DEGs in Nes-cKO neurons.

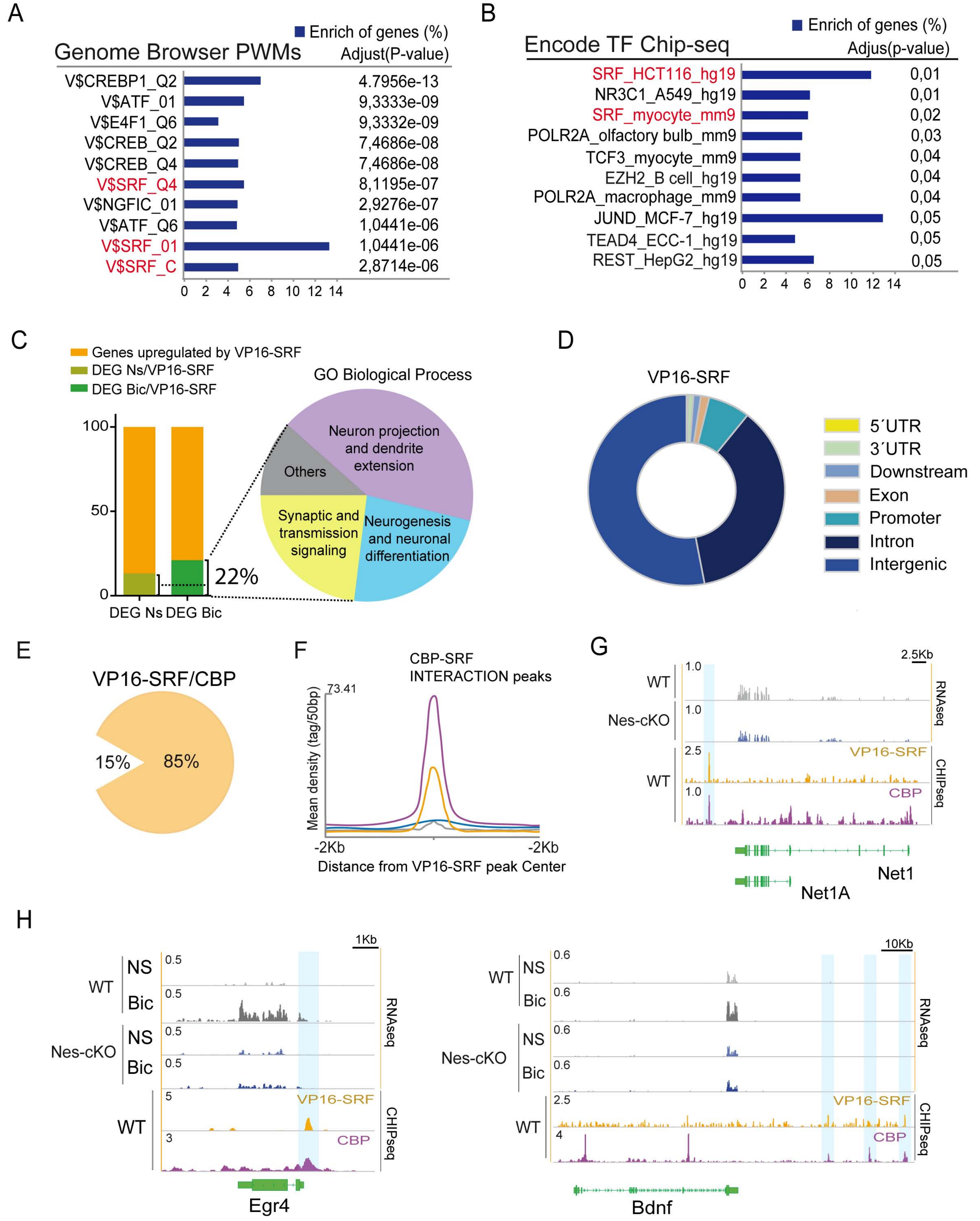
A
Enrich of genes (%)
Genome Browser PWMs
Adjust(P-value)
V$CREBP1_Q2 4.7956e-13
V$ATF_01 9,3333e-09
V$E4F1_Q6 9,3332e-09
V$CREB_Q2 7,4686e-08
V$CREB_Q4 7,4686e-08
V$SRF_Q4 8,1195e-07
V$NGFIC_01 2,9276e-07
V$ATF_Q6 1,0441e-06
V$SRF_01 1,0441e-06
V$SRF_C 2,8714e-06
0 2 4 6 8 10 12 14
B
Enrich of genes (%)
Encode TF Chip-seq
Adjus(p-value)
SRF_HCT116_hg19 0,01
NR3C1_A549_hg19 0,01
SRF_myocyte_mm9 0,02
POLR2A_olfactory bulb_mm9 0,03
TCF3_myocyte_mm9 0,04
EZH2_B cell_hg19 0,04
POLR2A_macrophage_mm9 0,04
JUND_MCF-7_hg19 0,05
TEAD4_ECC-1_hg19 0,05
REST_HepG2_hg19 0,05
0 2 4 6 8 10 12 14
C
Genes upregulated by VP16-SRF
DEG Ns/VP16-SRF
DEG Bic/VP16-SRF
GO Biological Process
100
50
0
DEG Ns DEG Bic
22%
Neuron projection and dendrite extension
Others
Synaptic and transmission signaling
Neurogenesis and neuronal differentiation
D
VP16-SRF
5´UTR
3´UTR
Downstream
Exon
Promoter
Intron
Intergenic
E
VP16-SRF/CBP
15%
85%
F
CBP-SRF INTERACTION peaks
73.41
Mean density (tag/50bp)
-2Kb
-2Kb
Distance from VP16-SRF peak Center
G
2.5Kb
WT
Nes-cKO
WT
1.0
1.0
2.5
1.0
RNAseq
VP16-SRF
CBP
CHIPseq
Net1
Net1A
H
1Kb
WT
NS
Bic
Nes-cKO
NS
Bic
0.5
0.5
0.5
0.5
RNAseq
WT
5
3
VP16-SRF
CBP
CHIPseq
Egr4
10Kb
WT
NS
Bic
Nes-cKO
NS
Bic
0.6
0.6
0.6
0.6
RNAseq
WT
2.5
4
VP16-SRF
CBP
CHIPseq
Bdnf

Inventory of Supplemental Tables

Table S1 related to Figure 4. Analysis of differentially expressed genes between genotypes (Nes-cKO *vs* cWT PNCs).

Table S2 related to Figure 4. Analysis of differentially expressed genes in cWT PNCs (non-stimulated *vs* Bic-stimulated).

Table S3 related to Figure 4. Analysis of differentially expressed genes in Nes-cKO PNCs (non-stimulated *vs* Bic-stimulated).

Table S4 related to Figure 5. VP16-SRF genome occupancy in the chromatin of hippocampal PNCs.
